# The impact of two state-level approaches to restricting the sale of flavored tobacco products

**DOI:** 10.1186/s12889-022-14172-y

**Published:** 2022-09-22

**Authors:** Tyra Satchell, Megan C. Diaz, Daniel Stephens, Adrian Bertrand, Barbara A. Schillo, Laurie P. Whitsel

**Affiliations:** 1 American Heart Association, Washington, DC USA; 2 Truth Initiative, Washington, DC USA

**Keywords:** Flavored tobacco products, Tobacco policy, Tobacco regulation

## Abstract

**Background:**

Flavored tobacco products are highly appealing to youth. The Federal government lacks a comprehensive flavored tobacco products policy and states have adopted different approaches restricting these products. This study analyzes the impact of Massachusetts’ comprehensive prohibition and New Jersey’s partial restriction on the sale of flavored tobacco products.

**Methods:**

NielsenIQ Retail Scanner data were used to construct four log per capita dependent variables: e-liquid milliliters, cigarette packs, cigars, and smokeless tobacco ounces for products flavored as fruit, menthol, mint, tobacco and other. All models used difference-in-differences regressions, with Virginia and Pennsylvania serving as controls. The models controlled for state level product prices, population percentages by race/ethnicity, proportion male, median household income, unemployment rate, minimum legal sales age, tobacco 21 policies, and cumulative cases and deaths of COVID-19; the models accounted for time-specific factors by using 4-week period fixed-effects.

**Results:**

There was a significant decrease in sales across all flavored tobacco products in Massachusetts, including fruit [-99.83%, *p* < 0.01], menthol [-98.33%, *p* < 0.01], and all other flavored [-99.28%, *p* < 0.01] e-cigarettes. The cigar group “all other-flavors" [-99.92%, *p* < 0.01] and menthol flavored cigarettes [-95.36%, *p* < 0.01] also significantly decreased. In New Jersey, there was a significant decrease in per capita sales of menthol-flavored e-cigarettes [-83.80%, *p* < 0.05] and cigar group “all other-flavors" experienced a significant increase in per capita sales [380.66%, *p* < 0.01].

**Conclusions:**

This study contributes to the growing body of evidence demonstrating the impact of sales prohibitions on reducing sales of flavored tobacco products. Statewide comprehensive approaches appear more effective than partial restrictions and should be prioritized.

**Implications:**

Results from this study support emerging research that demonstrates the promising effects of comprehensive flavoring sales prohibitions. This study can be used to inform future flavored tobacco product policy solutions developed by advocates and policy makers to curb overall tobacco initiation and use by youth and adults.

**Supplementary Information:**

The online version contains supplementary material available at 10.1186/s12889-022-14172-y.

## Introduction

The Family Smoking Prevention and Tobacco Control Act of 2009 banned characterizing flavors in cigarettes, but exempted menthol cigarettes and did not address flavors in other tobacco products [1]. In 2015, there were more than 250 unique cigar flavors on the United States (US) market [2] and as of 2017, there were over 15,000 e-liquid flavors being sold. Eighty one percent of youth ages 12 to 17 initiate with a flavored product [3]. Adult and youth tobacco users whose first use was a flavored tobacco product were more likely to use tobacco products regularly in the future [4]. In addition, the tobacco industry has historically promoted flavorings to Black communities and other disenfranchised groups [5, 6] and it is estimated that the continued presence of menthol cigarettes on the market has been a significant contributor for slowing the decline in smoking prevalence from 1980 to 2018 [7].

Flavorings in e-cigarettes, cigars, and other newer tobacco products have been a major contributor to their proliferation and epidemic of youth use [8]. In the United States in 2020, an estimated 3.65 million high school students and 800,000 middle school students indicated current use of any tobacco product, with e-cigarettes being the most commonly used tobacco product followed by cigars and cigarettes [9]. Nearly all (97%) of youth e-cigarette users reported current use of flavored e-cigarettes [10]. In January 2020, responding to the e-cigarette use-associated lung injury (EVALI) and the youth e-cigarette epidemic, the U.S. Food and Drug Administration (FDA) issued guidance [11] for the sale of some flavored e-cigarettes. The guidance prohibited companies from selling certain e-cigarettes with flavored cartridges that appeal to kids, including fruit and mint flavors. Companies that did not discontinue manufacturing, distributing, and selling the sale of flavored cartridges within 30 days risked enforcement action by the FDA [11]. However, e-liquids used in open-tank systems, all menthol and tobacco flavored e-cigarettes, and self-contained, disposable e-cigarettes were exempted from this guidance. Not surprisingly, surveillance has shown that youth quickly migrated to these exempted products [12, 13].

Some states and localities also responded by restricting the sale of flavored tobacco products and their components with different approaches, including adopting comprehensive sales prohibitions for all flavored products or partial sales restrictions removing flavored e-cigarettes only. Results from preliminary research have demonstrated that comprehensive sales prohibitions are likely to reduce the availability of flavored tobacco products in the retailer environment and decrease the use of tobacco products overall, as well as the use of flavored tobacco products [14, 15]. However, there has been a growing concern that partial flavoring sales restrictions may be less impactful and could result in increased use among other tobacco products, especially menthol cigarettes, and flavored cigars and cigarillos [16].

In November 2019, Massachusetts became the first state to pass a comprehensive law that immediately ended the retail sale of all flavored tobacco products, including flavored e-cigarettes, and menthol cigarettes. However, the law was only temporary. A less comprehensive, but permanent flavoring sales restriction was passed in December 2019 [17]. This law restricted the sale of flavored e-cigarette and combustible products, including menthol cigarettes and flavored chewing tobacco, to licensed smoking bars where they must be consumed on-site [17]. Beginning in June 2020, all retail outlets in Massachusetts were required to comply [17]. Comparatively, New Jersey implemented a partial sales restriction that went into effect April 2020 that prohibited the sale and distribution of flavored e-cigarettes and e-cigarette products, but excluded menthol cigarettes and other flavored combustible products [18]. Few studies have explored the impacts of both comprehensive and partial sales prohibitions on flavored tobacco products and regular tobacco product retail sales. This research project compared a comprehensive flavored tobacco product sales restriction to a less comprehensive flavored e-cigarette tobacco product sales restriction. The purpose of this paper was to analyze the impact of different policy strategies aimed to restrict the sale of flavored tobacco products in order to further inform policy approaches.

## Methods and data source

We used Nielsen Retail Scanner Sales data for e-cigarettes, cigarettes, cigars, and smokeless tobacco products licensed from NielsenIQ (Chicago, IL). Nielsen data were provided in aggregated 4-week periods and included sales from: participating independent, chain and gas station convenience stores; food, drug, and mass merchandisers; discount and dollar stores; and military commissaries. For this project we focused on data from Massachusetts (MA) (comprehensive sales prohibition) and New Jersey (NJ) (partial sales restriction) using Virginia (VA) and Pennsylvania (PA) as control states (no flavor tobacco product sales restrictions).

## Measures

### Dependent variables

We constructed four dependent variables: total per capita milliliters of e-liquid, total per capita standardized packs of cigarettes, total per capita cigar units, and total per capita smokeless tobacco ounces. For e-cigarettes we focused on products that contained e-liquid and excluded hardware, batteries, and starter kits with no e-liquid (3.8% of the data). We standardized cigarettes to packs of 20 units, cigars to total pieces of large cigars, tipped cigars, cigarillos, and little cigars, and total ounces for smokeless tobacco products. We defined smokeless tobacco products as snus, moist snuff, chewing loose-leaf, chewing plug twist, dry snuff, and nicotine powder pouches. We excluded smokeless tobacco products where ounce information was not available (0.7% of the data). We further categorized our dependent variables into flavor categories. E-cigarettes were categorized as fruit, menthol, tobacco, and other flavors. Cigarettes were categorized as tobacco and menthol flavors. Smokeless tobacco products were categorized as fruit, mint, and tobacco flavors; we excluded menthol (0.01% of the data) and other (5.3% of the data) flavored products. Cigars were categorized as fruit, tobacco, and other flavors. The category “other flavors” included flavors such as spices, desserts, coffee, and alcoholic beverages. For cigars, menthol (1.2% of the data) is included within other flavors. We utilized data aggregated to 4-week periods from the 4-week period ending on October 12, 2013 for e-cigarettes, cigarettes, and cigars to the 4-week period ending on September 5, 2020. For smokeless tobacco, we only had access to data from the 4-week period ending on November 8, 2014 to the 4-week period ending on September 5, 2020.

### Flavor restrictions

We estimated the impact of various flavor restrictions. In November 2019, MA passed its law ending the sale of all flavored tobacco products, including flavored e-cigarettes, menthol cigarettes and flavored cigars. The law immediately ended the sale of flavored e-cigarettes and starting June 1, 2020, the sale of flavored combustible cigarettes and other tobacco products were restricted to licensed smoking establishments [17]. To account for these different implementation dates, we created two dichotomous variables. The value “1” was used to indicate the presence of flavoring restrictions for e-cigarette products, starting with the 4-week period ending November 30, 2019. The second dichotomous variable was used for cigarette, cigar, and smokeless tobacco products and used the value “1” to indicate the presence of flavoring restrictions, starting with the 4-week period ending on July 11, 2020.

In January 2020, NJ signed legislation to impose a permanent ban on flavored e-cigarette products, which became effective on April 20, 2020 [18, 19]. For NJ the value, “1” was used to indicate the presence of flavor restriction regulations, starting with the 4-week period ending May 16, 2020. The states of VA and PA had no flavoring restrictions and were used as our control states.

### Control variables

We controlled, at the state level, for various population and policy variables. Included in our analysis were population percentages by race/ethnicity (non-Hispanic White, non-Hispanic Black, non-Hispanic Others, Hispanic), and sex. The analysis also controlled for inflation adjusted median household income (September 2020 dollars) and state unemployment rates [20–22]. We also controlled for minimum legal sales age and state tobacco 21 policies. These data were identified from the Centers for Disease Control and Prevention (CDC) State Tobacco Activities Tracking and Evaluation (STATE) System [23]. To indirectly control for excise and wholesale percentage taxes, we used Nielsen data to construct inflation adjusted weighted average prices for one milliliter of e-liquid, a standardized pack of 20 cigarettes, one cigar piece, and one ounce of smokeless tobacco (September 2020 dollars). Lastly, we also used Nielsen data to construct and control for various product market shares. For our e-cigarette regressions we controlled for the market share of disposable products; for cigars we controlled for the market share of cigarillos and large cigars; and for smokeless tobacco we controlled for the market share of snus and moist snuff.

To account for the novel coronavirus (COVID-19) pandemic, we used cumulative cases and deaths from the CDC in each state [24]. Finally, to account for time-specific factors, the analysis controlled for 4-week period time fixed effects.

### Statistical analysis

For all our analysis we used difference-in-differences (DID) regression models to evaluate MA full product sales flavor prohibition, and NJ e-cigarette flavor restriction on fruit-, tobacco-, menthol-, and other-flavored e-cigarette; menthol and tobacco cigarettes; fruit-, mint-, and tobacco-flavored smokeless tobacco sales; and fruit-, tobacco-, and other-flavored cigars sales [25, 26]. We used data from VA and PA as control states in our models. Our dependent variables were log per capita total e-liquid milliliters sold, log per capita total standardized cigarette packs sold, log per capita total cigar units sold, and log per capita total smokeless tobacco ounces for each of the above-mentioned flavors. For all models we ran a parallel trends assumption tests on the pre-treatment data. The analysis was conducted using Stata version 17.

## Results

Table 1 presents summary statistics of control variables for our samples from MA, NJ, VA and PA for the overall time-period for each respective state. Supplemental Table 1 presents summary statistics for the pre-implementation periods of all of control variables for our samples. DID results will be more plausible if levels and trends of the control and treatment groups are similar during the pre-implementation period. All states had similar median household incomes, minimum sales age, and demographic information. However, Virginia had lower prices and higher per capita sales across all tobacco products, excluding cigars, among all four states, while NJ, PA, and MA were more comparable. Additionally, NJ reported the highest number of COVID cases [11,951] compared to VA [4,885], PA [6,544] and MA [7,370] across all time periods. The states chosen as controls, PA and VA, were similar to our treatment states, MA and NJ prior to the flavor bans.

**Table 1:** Summary statistics of key variables, demographic characteristics, state-level policy controls, and Covid 19 controls at all time periods

	All time periods				
	**Massachusetts All time Periods**	**New Jersey All time Periods**	**Virginia All time Periods**	**Pennsylvania All time Periods**	**Average of Virginia and Pennsylvania All time Periods**
***Outcome Variables***					
Per Capita E-Cigarette (Milliliters of E-Liquid)	0.02 [0.02]	0.04 [0.04]	0.02 [0.04]	0.03 [0.03]	0.03 [0.04]
Per Capita Cigarette (Packs of Cigarettes)	0.95 [0.48]	1.30 [0.34]	2.15 [0.41]	1.69 [0.27]	1.92 [0.42]
Per Capita Smokeless Tobacco (Ounces)	0.03 [0.03]	0.05 [0.04]	0.22 [0.15]	0.22 [0.14]	0.22 [0.14]
Per Capita Cigar (Pieces)	0.28 [0.22]	0.33 [0.25]	0.54 [0.36]	0.35 [0.24]	0.44 [0.32]
***Demographic Characteristics***					
Non-Hispanic White	0.73 [0.00]	0.56 [0.01]	0.62 [0.00]	0.77 [0.01]	0.70 [0.07]
Non-Hispanic Black	0.07 [0.00]	0.13 [0.00]	0.19 [0.00]	0.11 [0.00]	0.15 [0.04]
Non-Hispanic Other	0.09 [0.00]	0.11 [0.00]	0.09 [0.00]	0.05 [0.00]	0.07 [0.02]
Hispanic	0.12 [0.00]	0.20 [0.00]	0.09 [0.00]	0.07 [0.00]	0.08 [0.01]
Proportion Male	0.48 [0.00]	0.48 [0.00]	0.48 [0.00]	0.48 [0.00]	0.49 [0.00]
***State-level Policy Controls***					
Real median household income	$75,834 [7,758]	$72,693 [6,891]	$70,447 [6,651]	$62,187 [4,008]	$66,317 [6,870]
Unemployment rate	4.66 [2.76]	5.43 [2.95]	4.00 [1.60]	5.62 [2.28]	4.81 [2.13]
Minimum legal e-cigarette sales age	18.11 [0.32]	19.95 [1.00]	18.60 [1.20]	18.11 [0.57]	18.36 [0.97]
Minimum legal cigarette sales age	18.11 [0.32]	19.98 [1.00]	18.60 [1.20]	18.11 [0.57]	18.36 [0.97]
Tobacco 21 laws	0.29 [0.45]	0.48 [0.50]	0.20 [0.40]	0.04 [0.19]	0.12 [0.32]
Real weighted average e-cigarette ml price	$8.69 [6.61]	$9.21 [11.80]	$6.71 [1.35]	$9.41 [2.59]	$8.06 [2.46]
Real weighted average cigarette pack price	$8.60 [0.61]	$7.53 [0.63]	$4.82 [0.46]	$6.59 [0.88]	$5.70 [1.14]
Real weighted average smokeless tobacco ounce price	$8.53 [1.29]	$4.28 [0.52]	$2.65 [0.35]	$2.92 [0.66]	$2.79 [0.54]
Real weighted average cigar piece price	$0.69 [0.16]	$0.67 [0.04]	$0.55 [0.06]	$0.52 [0.05]	$0.53 [0.06]
***Market Shares***					
Disposable e-cigarettes	0.19 [0.14]	0.14 [0.09]	0.12 [0.09]	0.12 [0.09]	0.12 [0.09]
Cigarillos	0.68 [0.03]	0.78 [0.03]	0.80 [0.03]	0.81 [0.03]	0.80 [0.03]
Large Cigars	0.22 [0.05]	0.16 [0.03]	0.16 [0.02]	0.13 [0.03]	0.14 [0.03]
Snus	0.36 [0.05]	0.47 [0.04]	0.24 [0.02]	0.31 [0.02]	0.28 [0.03]
Moist Snuff	0.61 [0.04]	0.50 [0.04]	0.67 [0.02]	0.66 [0.02]	0.66 [0.02]
***Covid-19***					
Cumulative Cases	7,370 [27,248]	11,951 [43,262]	4,885 [20,389]	6,544 [24,964]	5,714 [22,792]
Cumulative Deaths	512 [1,938]	860 [3,179]	122 [484]	418 [1,607]	270 [1,195]

### Difference-in-differences results

Table 2 presents our results from our various flavored-tobacco products DID models. Results demonstrate a significant decrease in sales in MA and NJ in most of the restricted products following policy implementation. However, the degree of reduction varies by tobacco product, flavor type, and type of policy. Following implementation, Massachusetts (Supplemental Fig. 1), a state with a more comprehensive sales prohibition, saw significant decreases across all flavored tobacco products. Most notably, fruit [-99.83%, *p* < 0.01], menthol [-98.33%, *p* < 0.01], all other [-99.28%, *p* < 0.01], and to a lesser degree tobacco flavored [-81.18%, *p* < 0.05] e-cigarette flavorings had a significant reduction in per capita sales. The cigar group “all other-flavors" [-99.92, *p* < 0.01] and menthol flavored cigarettes [-95.36%, *p* < 0.01] also had a significant decrease in MA (Supplemental Fig. 1). After implementation of its partial sales restriction New Jersey (Supplemental Fig. 2) only experienced a significant decrease in per capita sales of menthol-flavored e-cigarettes [-83.80%, *p* < 0.05]. Additionally, the cigar group “all other-flavors" experienced a significant increase in per capita sales [380.66, *p* < 0.01]. There were no other statistically significant increases or decreases in other products in NJ following implementation. Supplemental Fig. 3 illustrates per capita tobacco product unit sale averages for our control states, PA and VA.

**Table 2:** Adjusted per capita % change per time unit^a^ (Standard Errors)^h^

	**Massachusetts**^**b**^** (Nov 30, 2019**^**c**^**-Sep 5, 2020)**	**New Jersey**^**b**^** (April 19, 2020**^**d**^**-Sep 5, 2020)**
***Per Capita E-Cigarette (Milliliters of E-Liquid)***		
* Fruit-flavored e-cigarettes*	-99.83^***^ (0.208)	-82.27^**f**^ (0.788)
* Tobacco-flavored e- cigarettes*	-81.18^**^ (0.219)	7.25 (0.362)
* Menthol-flavored e- cigarettes*	-98.33^***^ (0.403)	-83.80^**^ (0.301)
* All other-flavors e- cigarettes*	-99.28^***f^ (0.374)	-86.47 (0.753)
	**Massachusetts**^**b**^** (July 11, 2020**^**e**^**-Sep 5, 2020)**	**New Jersey**^**b**^** (April 19, 2020**^**d**^**-Sep 5, 2020)**
***Per Capita Cigarette (Standardized Packs)***		
* Menthol-flavored cigarettes*	-95.36^***^ (0.126)	-10.42^**f**^ (0.203)
* Tobacco-flavored cigarettes*	-22.12^*f^ (0.071)	-13.06^**f**^ (0.274)
***Per Capita Smokeless Tobacco (Ounces)***		
* Fruit-flavored smokeless tobacco*	-13.93^**f**^ (3.129)	-33.63^**f**^ (0.476)
* Mint-flavored smokeless tobacco*	-60.94^**f**^ (2.521)	-24.42^**f**^ (0.187)
* Tobacco-flavored smokeless tobacco*	115.98^**f**^ (0.375)	-3.92^**f**^ (0.388)
***Per Capita All Cigars (Pieces)***		
* Fruit-flavored cigars*	-95.45^**f^ (0.458)	185.77 (1.136)
* Tobacco-flavored cigars*	215.82^**f**^ (0.238)	452.90 (1.122)
* All other-flavored cigars*	-99.92^***f^ (0.530)	380.66^***^ (0.086)

^***^p < 0.01

^**^p < 0.05

^*^p < 0.1

^a^Time unit is by 4-week period

^b^Pennsylvania and Virginia are the control states in each regression

^c^Effective date December 11, 2019

^d^Effective date April 20, 2020

^e^Effective date June 1st, 2020

^f^Parallel-trends test support parallel-trends assumption (*p* > 0.05)

^h^All models use difference-in-differences (DID) regression methodology. All models controlled for real product prices, population percentages by race/ethnicity, proportion males, real median household income, state unemployment rate, minimum legal sales age, state tobacco 21 policies, and state cumulative cases and deaths of COVID-19. E-cigarette models include market share of disposable products; cigar models include market share of cigarillos and large cigars; and smokeless tobacco models include market share of snus and moist snuff. Lastly each model accounts for time-specific factors by using 4-week period time fixed effects. Point estimates are reported as percentage changes

## Discussion

States and localities have an important role in strengthening or addressing gaps in federal regulation or legislation, including around flavored tobacco products. As of March 2022, approximately 64,828,574 people were covered by 361 state or local flavored tobacco sales restrictions, representing 20% of the US population [27]. This analysis builds on the evidence base that comprehensive approaches to restricting flavored tobacco products provide an effective policy approach to reducing sales of all flavored products with a future potential to impact population health more positively [15]. In our research we found that in Massachusetts, where a more comprehensive sales prohibition was implemented, sales of fruit-, menthol-, tobacco-, and all other-flavored e-cigarettes decreased substantially, consistent with prior research performed for Massachusetts [28, 29] and Lowell, Massachusetts [30, 31]. In addition to decreases in e-cigarette per capita sales, we also observed decreases in menthol-flavored cigarettes and fruit- and other-flavored cigars. These reductions stand in stark contrast to the limited decreases observed in New Jersey, where only menthol-flavored e-cigarettes decreased. The differences observed further illustrate the importance of comprehensive flavor laws in the near term that can lead to decreases in youth use one to two years post implementation [32].

State and local approaches to flavored tobacco product restrictions are taking place within a larger ecosystem of FDA regulatory action and industry innovation. FDA is deciding on pre-market tobacco product applications and substantial equivalence reports for “new” tobacco products that were not on the market or have been modified since February 15, 2007. To date, FDA has denied marketing orders for almost all flavored products, but as of June 2022 is still deciding on products with the most significant market share. Previously, in some cases, industry was responding to these denials by reformulating their products with synthetic nicotine.

On March 15, 2022, federal legislation closed that loophole in a legislative provision amending the definition of “tobacco product” as “any product made or derived from tobacco, or containing nicotine from any source, that is intended for human consumption,” giving FDA authority to regulate these products. After July 13, 2022, any synthetic nicotine product not authorized by FDA must come off the market. It will be important for states and local jurisdictions to reinforce FDA regulation in this area.

In April 2022, the FDA issued a proposed rule to eliminate menthol as a characterizing flavor in cigarettes and prohibit all flavored cigars [33]. Our findings for MA highlight the importance of comprehensive sales prohibitions to avoid product substitution. This is reflected in the recently proposed FDA rule to eliminate menthol from cigarettes while also removing flavored cigars from the marketplace. However, the final rule-making process will take time and will be subject to significant legal challenges from industry. Accordingly, states and local jurisdictions will continue to have an important role in eliminating the sales of all flavored tobacco products from the marketplace.

There are some limitations of this research. First, sales data are not a direct measure of tobacco use prevalence. Also, these sales data do not reflect online purchasing or purchasing from vape shops, which can account for an estimated one third of all e-cigarette market sales [31]. Currently, there is no publicly available database that effectively tracks these sales. Purchasing from neighboring states, with less restrictive flavoring policies, was not captured in the data. To properly account for the sale of e-cigarette products, we used per capita ml of e-liquid. Unfortunately, we did not have complete data for earlier 4-week periods. While this may affect early 4-week time periods, we had close to 90% of all milliliter information at the time of treatment. Finally, while we attempted to control for a large range of tobacco endgame policies, not all our DID models met the parallel-trends assumption. Given that a majority of our models were robust, we believe our models support our findings, while acknowledging that, future research should focus on using more than two control states to evaluate the effect of these policies and to provide more robust parallel trends between treatment and control states.

Additional research should explore tobacco retailer compliance with structure and implementation of flavor prohibitions, as well as the effect on youth flavored tobacco use. A comprehensive qualitative review [34] of studies done to date has shown that more robust study designs and methods and longitudinal follow-up are needed to continue to assess population health impact for policy implementation and outcome evaluation around flavored tobacco sales restrictions. Evaluation of different policies at the state and local levels, in combination with federal regulation, can provide insights into optimal structures and interactions of tobacco endgame policies for optimal health impact.

## Conclusions

This study supports emerging evidence that demonstrates the promising effects of comprehensive flavoring sales prohibitions. Partial restrictions may lead to increased uptake in flavored combustible tobacco products with serious potential implications for population health. Statewide comprehensive tobacco flavor sales prohibitions appear to be more impactful than e-cigarette-specific flavored policies in reducing flavored tobacco purchasing overall and therefore, should be prioritized by advocates and policy makers at all levels of government.

## Supplementary Information

**Additional file 1.** 

**Additional file 2.** 

**Additional file 3.** 

**Additional file 4.** 

## Data Availability

Due to contractual agreements, data cannot be made available to the public. The data that support the findings of this study are available from Nielsen but restrictions apply to the availability of these data, which were used under license for the current study, and so are not publicly available. Data are however available from the authors upon reasonable request and with permission of Nielsen.
