# Supplementary material for: The impact of two state-level approaches to restricting the sale of flavored tobacco products
Source: BMC Public Health. 2022 Sep 22;22:1799. doi: 10.1186/s12889-022-14172-y (PMC9493160; doi:10.1186/s12889-022-14172-y)
Supplement: Supplementary file 1 — Additional file 1. [file 12889_2022_14172_MOESM1_ESM.docx]

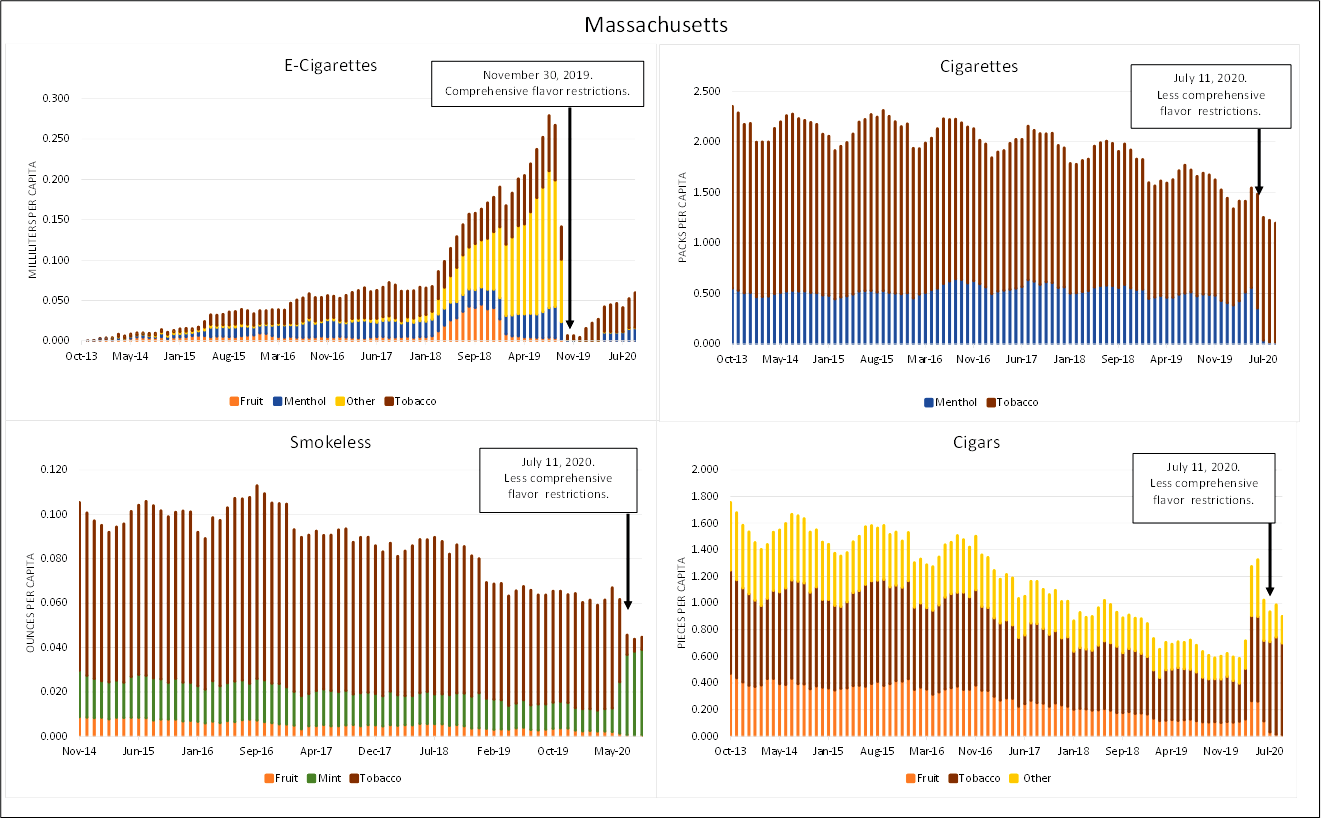


Figure 1: Massachusetts per capita tobacco product unit sale. Following implementation of a comprehensive flavor sales prohibition, the state of Massachusetts saw significant decreases across all flavored tobacco products sales.
