# Supplementary material for: The impact of two state-level approaches to restricting the sale of flavored tobacco products
Source: BMC Public Health. 2022 Sep 22;22:1799. doi: 10.1186/s12889-022-14172-y (PMC9493160; doi:10.1186/s12889-022-14172-y)
Supplement: Supplementary file 2 — Additional file 2. [file 12889_2022_14172_MOESM2_ESM.docx]

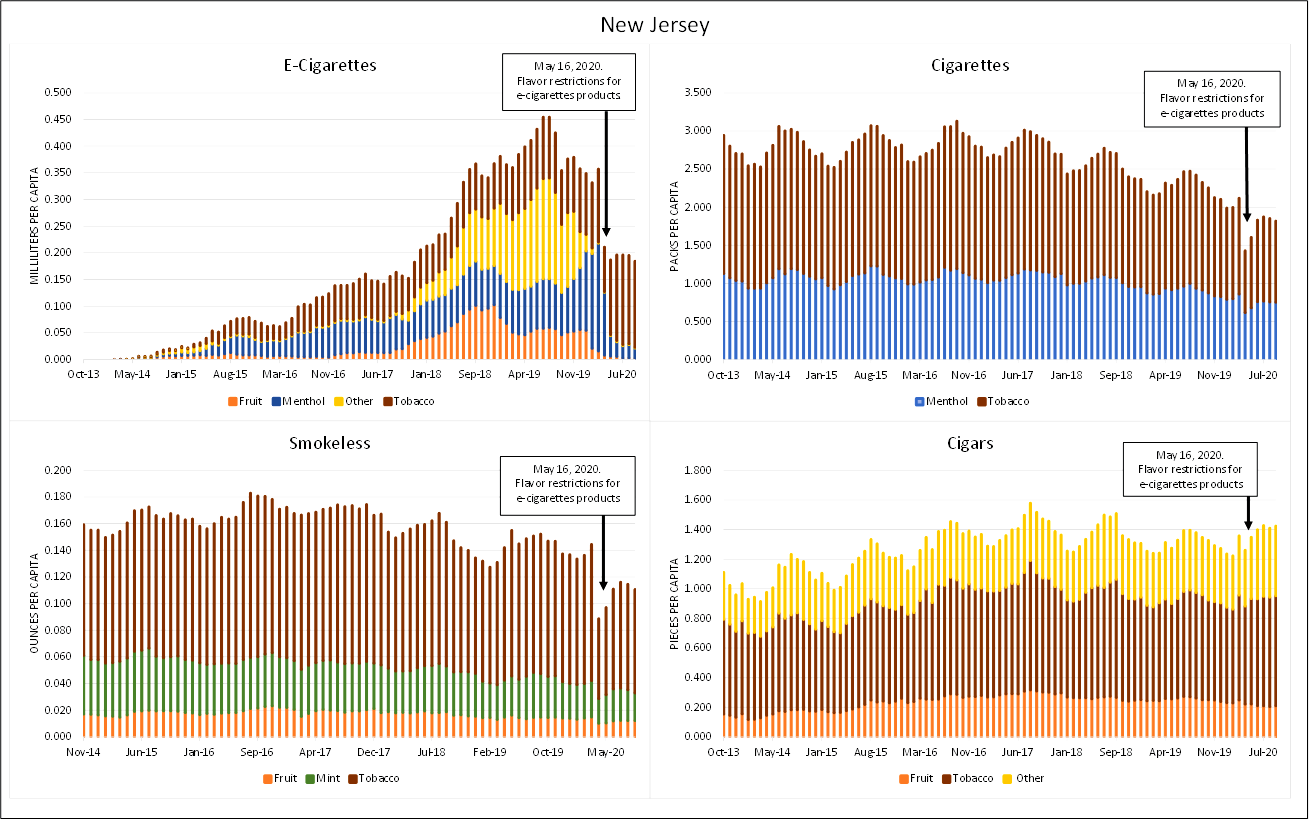


Figure 2: New Jersey per capita tobacco product unit sale. After implementation of a partial flavor sales restriction, the state of New Jersey only experienced a significant decrease in per capita sales of menthol-flavored e-cigarettes [-83.80%, p<0.05]. Additionally, the cigar group “all other-flavors" experienced a significant increase in per capita sales [380.66, p<0.01].
