# Supplementary material for: The impact of two state-level approaches to restricting the sale of flavored tobacco products
Source: BMC Public Health. 2022 Sep 22;22:1799. doi: 10.1186/s12889-022-14172-y (PMC9493160; doi:10.1186/s12889-022-14172-y)
Supplement: Supplementary file 3 — Additional file 3. [file 12889_2022_14172_MOESM3_ESM.docx]

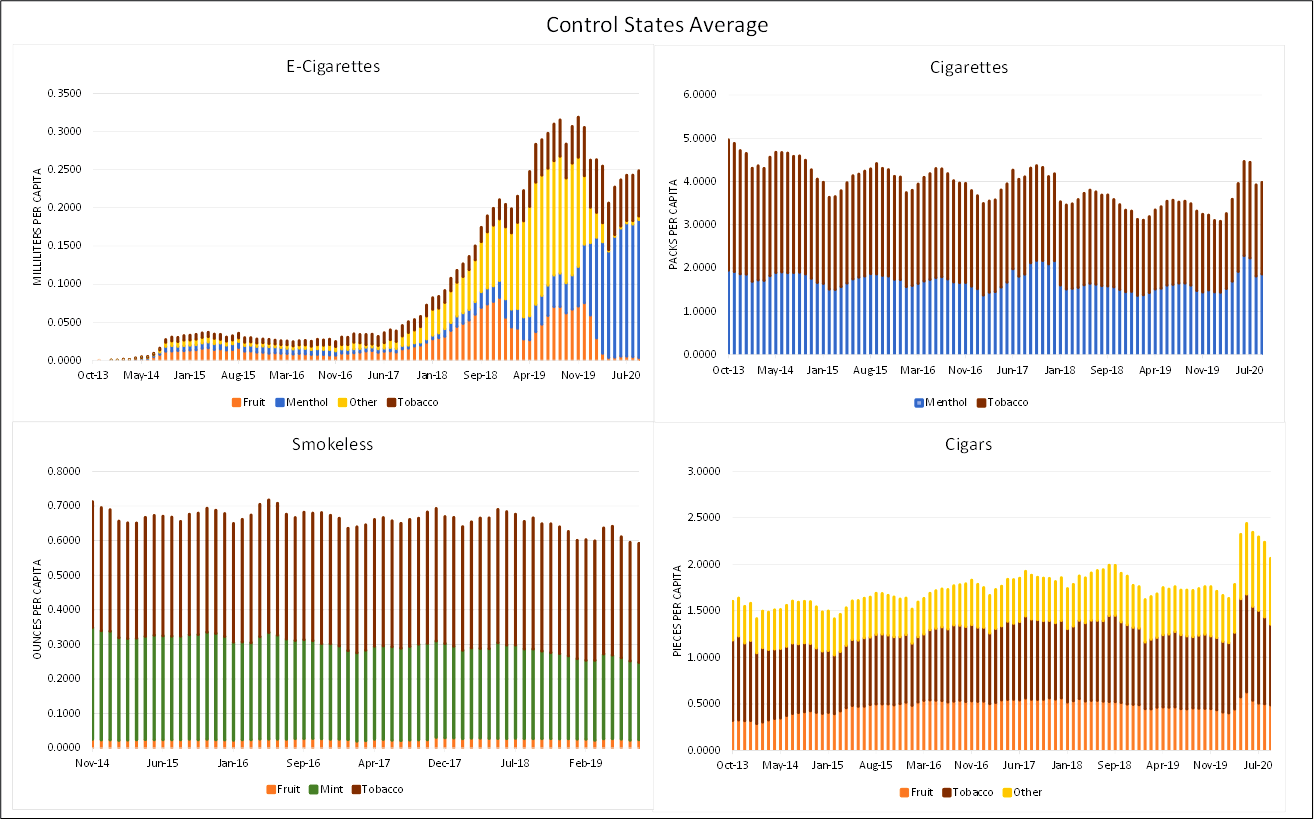


Figure 3: Control States. The figure illustrates the per capita tobacco product unit sale averages for our control states, Pennsylvania and Virginia.
